# Authors’ Response to Peer Reviews of “SARS-CoV-2 Vaccination Uptake in a Correctional Setting: Cross-sectional Study”

**DOI:** 10.2196/31900

**Published:** 2021-09-28

**Authors:** Justin Berk, Matthew Murphy, Kimberly Kane, Philip Chan, Josiah Rich, Lauren Brinkley-Rubinstein

**Affiliations:** 1 Rhode Island Department of Corrections Cranston, RI United States; 2 Rhode Island Department of Corrections Providence, RI United States; 3 Warren Alpert Medical School Brown University Providence, RI United States; 4 Rhode Island Department of Health Providence, RI United States; 5 Chapel Hill School of Medicine University of North Carolina Chapel Hill, NC United States

**Keywords:** vaccination, COVID-19, incarcerated individuals, correctional facility, public health, pandemic, vaccine, carceral setting, vaccine implementation, correctional staff

*This is the authors’ response to peer-review reports for the paper “SARS-CoV-2 Vaccination Uptake in a Correctional Setting”.*

## Round 1 Review

The authors of the manuscript [1] are grateful to the editor and reviewers [2,3] for their invaluable input and feedback.

### Anonymous [2]

#### General Comments

Thank you. We agree this is an important contribution.

#### Specific Comments

##### Introduction

Thank you for this comment. The first vaccine was administered on December 22, 2020, which, to our knowledge, was the first. At the beginning of the submissions process (at the time of the preprint server submission), this was meant to showcase that correctional facilities could and are offering vaccines. Now, as the vaccine is more widely available, we agree that there is less value added to showcasing Rhode Island as “the first,” and, therefore, this has been removed as suggested. The study period was made clearer as suggested as well.

##### Methods

1. This sentence (“From the beginning of the pandemic…”) has been moved to the Background section.

2. We have added a line that the Rhode Island Department of Corrections (RIDOC) leadership prioritized vaccine allocation based on guidance from the Centers for Disease Control and Prevention (CDC) and the Department of Health.

3. This change was made.

4. We agree this term is unhelpful and informal. We have changed “round” to “phase” to refer to all subsequent vaccination groups.

5. Thank you for identifying this confusion. The line has been rewritten to say: “This vaccine campaign exemplified adherence to public health principles: vaccinate where spread and disease can best be prevented.” A citation was added to clarify.

6. We agree these details can be important. We have added specifics that the education during roll call addressed information on signing up and have added a link to the video and uploaded the email as a supplement.

##### Results

1. This sentence has been moved to the Discussion.

2. The word “approximately” has been removed.

3. The details on uptake have been removed from the text, which now references only the table.

4. Details on second doses have been removed as suggested.

5. We agree this is an important finding and is now the topic sentence of its own paragraph.

6. An overpull is the phenomenon that most 10-dose vials actually had 11 or 12 doses that could be used, which was recommended by the CDC. This caused some headaches in logistics planning. This has therefore been left in but with a parenthetical explanation: “During this time “overpulls” (ie, a common 11th dose of vaccine could be pulled from a 10-dose vial)…”

7. Thank you for this clarification. Typically, it is capitalized when referring to the specific facility (ie, Intake facility) as opposed to a general intake facility.

8. The section was removed. We followed all Vaccine Adverse Event Reporting System (VAERS) protocols for tracking adverse events but had none, and this may therefore take away from the core part of the results.

##### Discussion

1. Agreed. We have removed the efficiency description and appreciate this feedback.

2. Agreed. These have now been split into two sentences, and we agree that they read much more clearly now.

3. As mentioned above, we have removed the discussion on the RIDOC being the first to vaccinate. We appreciate this feedback.

4. They were not. It is unclear and is most likely due to cultural issues in each facility. This would be a great topic for another paper. The Women’s Facility, on average, does have a shorter length of stay than the other sentenced facilities, but identifying factors of vaccine hesitancy among our own population is a topic of future research.

5. Thank you for this; we appreciate it. We used “difficult to reach” to refer to the overall demographics of individuals with limited access or uptake of vaccines, which often refers to BIPOC (black, indigenous, and other people of color) communities, which are also disproportionately affected by mass incarceration. Clearly, however, we would not want this to be misconstrued in any way and so “difficult to reach” has been removed from the manuscript.

6. Agreed. We have put less emphasis on switching to the single dose, particularly now with the complications of the Johnson & Johnson vaccine. Single-dose vaccines, however, do play a role in larger, short-term, jail-like facilities, and this was more explicitly said. The low second-dose refusal rate likely corresponds to community averages, although I do not believe there is strong data on this currently, and we, unfortunately, do not have detailed data explaining the reasons for refusals of the second dose. We agree this would be another wonderful future topic of research.

### Reviewer B

#### General Comments

We appreciate this opportunity to clarify and have removed the term “evaluation” and added a section regarding the RIDOC that I believe makes the writing clearer. Thank you for this feedback.

#### Specific Comments

##### Major/Minor Comments

###### Introduction

1. This is very reasonable, and we appreciate the critique. This language has been changed to state: “Correctional outbreaks have been shown to contribute to the community and statewide spread of infection.”

2. We agree. The term “evaluation” has been removed.

###### Methods

1. The term “aggressive” has been removed.

2. We have worked to make referencing “the RIDOC” more consistent as colloquially it is referred to both as “RIDOC” and as “the RIDOC.” It is now referred to consistently as “the RIDOC” when used as a noun or as “RIDOC” when used as an adjective (eg, “RIDOC nurses”). We have changed the wording to better define “security facility” and now consistently refer to the group of individuals as “sentenced individuals.”

We have added a description: “The Rhode Island Department of Corrections (RIDOC) is a unified (combined prison and jail) statewide correctional facility that currently houses approximately 1500 sentenced and 500 awaiting-trial individuals across 6 facilities among a spectrum of security levels, including Minimum Security, Medium Security, Maximum Security, and High Security.)”

3. This sentence has been taken out as the majority of this paper focuses on the sentenced population. A better description of the Intake facility is included.

4. The term “rounds” has been replaced by “phases” as mentioned above. The term “opt-out” was removed, and a better description of the public health educators is included. Most of the education was tailored to the individual, and so we have added a statement regarding answering questions. This is now described as: “Two RIDOC public health educators provided education on the vaccine, answered questions, and provided consent before the vaccine clinic day. All eligible individuals were offered the vaccine in this way with the option to accept or defer.”

5. Thank you for identifying this. We now explicitly state the names of each smaller facility in the text: “In phase 2, smaller facilities (ie, facilities with a smaller average daily population: Women’s Facility; Minimum, Maximum, and High Security facilities) were offered the vaccine…”

6. Thank you for this opportunity to clarify. We have changed the wording to explain opt-in via email: “Among corrections staff, individuals were vaccinated with an opt-in system (signing up via email).”

###### Results

1. The parentheses have been removed.

2. We have added the article. Thank you for catching this.

3. Thank you, this wording has been changed as suggested.

4. This sentence has been removed to avoid confusion.

5. This section was removed and now references Table 1 (as recommended by Anonymous).

###### Discussion

1. We have removed the term “efficient,” as also recommended by Anonymous.

2. This is appreciated and was also suggested by Anonymous. The change has been made to split this into two sentences: “This aligns with necessary immunization rates modeled to achieve herd immunity [8]. More importantly, this is a departure from some concerns of high vaccine hesitancy rates, including a recent CDC publication estimating only a 45% willingness to receive the vaccine among incarcerated people [9].”

3. The term “devastated” has been removed to avoid editorializing.

4. This sentence has been changed to say, “Additionally, both COVID-19 and mass incarceration have disproportionately impacted communities of color [11].” We have made changes to consistently use “Covid-19” rather than “COVID-19,” although we also defer to the journal’s editorial preference.

##### Tables

1. Table 1: Thank you for identifying this. This is now clarified in the text to align with the table.

2. Table 1: The asterisk (regarding the type of vaccine used) has been removed and added to the text.

3, 4. Table 2: The reviewer is completely correct that the Intake population, being more jail-like, adds some confusion to the paper and takes awareness from the core focus, which was on the immediate vaccination of sentenced individuals (some of whom just happened to be at our jail-like Intake facility). Table 2, therefore, has been removed, as it does not further elaborate on the key findings of the research and only adds questions.
